# Exploring the Antimalarial Potential of *Gnetum gnemon* Leaf Extract Against *Plasmodium berghei* in Mice

**DOI:** 10.1155/jotm/3471083

**Published:** 2024-11-23

**Authors:** Sakaewan Ounjaijean, Voravuth Somsak

**Affiliations:** ^1^Research Institute for Health Sciences, Chiang Mai University, Chiang Mai 50200, Thailand; ^2^School of Allied Health Sciences, Walailak University, Nakhon Si Thammarat 80160, Thailand; ^3^Research Excellence Center for Innovation and Health Products, Walailak University, Nakhon Si Thammarat 80160, Thailand

**Keywords:** antimalarial potential, *Gnetum gnemon*, in vivo study, *Plasmodium berghei*

## Abstract

Malaria remains a critical global health issue, particularly in tropical and subtropical regions. The disease, caused by *Plasmodium* parasites, is transmitted by *Anopheles* mosquitoes and can lead to severe complications and death if untreated. The emergence of drug-resistant strains highlights the urgent need for new antimalarial agents. *Gnetum gnemon*, a plant native to Southeast Asia, has shown promise due to its rich bioactive compounds. This study aims to evaluate the suppressive, curative, and prophylactic antimalarial potential of *Gnetum gnemon* leaf extract (GGE) against *Plasmodium berghei* in mice. GGE was prepared using a combination of hot water extraction and microwave-assisted heating. Acute toxicity tests revealed no significant adverse effects at a dose of 3000 mg/kg. The doses of 100, 200, and 400 mg/kg were selected based on preliminary toxicity assessments to systematically investigate the dose-dependent antimalarial efficacy of the extract. Suppressive tests showed that GGE at doses of 100, 200, and 400 mg/kg significantly reduced parasitemia levels, with the highest dose achieving a 63.97% inhibition. In these tests, GGE also increased the mean survival time (MST) of treated mice compared to untreated controls. However, GGE did not exhibit significant curative effects, as parasitemia levels in the treated groups were similar to the untreated control group. Prophylactic tests indicated that GGE pretreatment did not significantly reduce parasitemia levels or improve MST compared to controls, unlike chloroquine (CQ), which demonstrated potent prophylactic efficacy with a significant increase in MST. These findings suggest that while GGE has notable suppressive antimalarial activity, it does not exhibit strong curative or prophylactic effects at the tested doses. This study contributes to the understanding of plant-based antimalarial agents and underscores the importance of continued exploration of natural products for malaria treatment.

## 1. Introduction

Malaria remains one of the most significant global health challenges, particularly affecting tropical and subtropical regions. The disease is caused by *Plasmodium* parasites, with *Plasmodium falciparum* being the most deadly species. Transmitted through the bites of infected *Anopheles* mosquitoes, malaria manifests with symptoms ranging from fever, chills, and flu-like illness to severe complications such as anemia, cerebral malaria, and multiorgan failure [1]. Despite substantial progress in reducing malaria incidence and mortality through interventions such as insecticide-treated bed nets, antimalarial drugs, and indoor residual spraying, malaria continues to exact a heavy toll, particularly in sub-Saharan Africa where the majority of cases and deaths occur [2]. The emergence of drug-resistant malaria strains poses a significant threat to these gains, underscoring the urgent need for novel antimalarial agents and treatment strategies [3]. Effective control and eventual eradication of malaria will require sustained investment in research and development, improved access to existing interventions, and the integration of new, innovative approaches, including the exploration of plant-based therapeutics.

**Figure 1:**
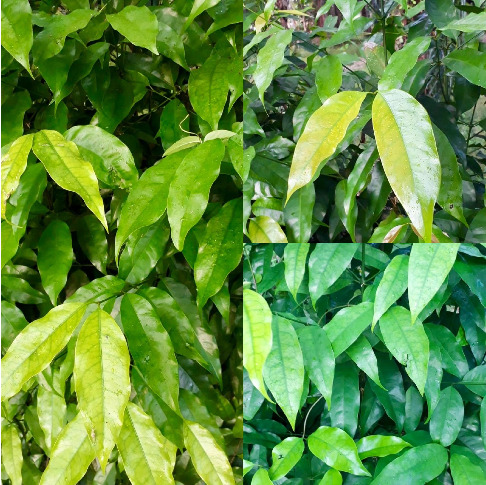
Picture of *Gnetum gnemon* leaf.

*Gnetum gnemon* (Figure 1), a perennial plant commonly found in Southeast Asia, has garnered significant attention in recent years due to its diverse medicinal properties. Traditionally used in various cultures for its nutritional and therapeutic benefits, *G. gnemon* is rich in bioactive compounds such as flavonoids, tannins, and phenolic compounds. Recent research has highlighted its potential in addressing a range of health issues, including its antioxidant, anti-inflammatory, and antimicrobial activities [4]. Notably, studies have begun to explore its antimalarial properties, revealing promising results against *Plasmodium* species. For instance, extracts from *G. gnemon* have demonstrated significant inhibitory effects on *P. falciparum* in vitro [5]. However, despite this encouraging finding, there is a notable lack of in vivo research evaluating the antimalarial efficacy of *G. gnemon* in animal models. Hence, this study aims to fill this gap by systematically investigating the suppressive, curative, and prophylactic antimalarial activities of *G. gnemon* leaf extract against *Plasmodium berghei* in mouse model. By doing so, we seek to provide comprehensive insights into the potential of *G. gnemon* as a novel antimalarial agent and contribute to the development of effective plant-based therapeutics for malaria.

## 2. Materials and Methods

### 2.1. Preparation of Extraction

*Gnetum gnemon* leaves were obtained from the local market at the South of Thailand during the rainy season (May to August), and identification was made by researcher at Faculty of Pharmacy, Western University. The voucher specimen was numbered (GGE-WTU/068) and deposited at Western University. The leaf extract of *Gnetum gnemon* was prepared using a method combining hot water extraction and microwave-assisted heating [6]. Prior to extraction, the collected leaves were thoroughly washed with distilled water (DW) to remove debris and contaminants. After washing, the leaves were air-dried in the shade with temperature of 25°C until they reached a constant weight. Subsequently, the dried leaves were ground into a fine powder using an electric blender. For the extraction process, the powdered leaves were mixed with DW at a concentration of 10% (w/v). The mixture underwent microwave-assisted heating at 500 W for 5 min to facilitate efficient extraction of bioactive compounds. Following microwave treatment, the extract was filtered using Whatman no. 1 filter paper to remove particulate matter and then concentrated using lyophilization to obtain a dry powder form (11.46 ± 0.016% yield) of *G. gnemon* extract (GGE). The extract was stored at −20°C until used.

### 2.2. Experimental Mice

Male ICR mice, aged 4–6 weeks and weighing 20–25 g, were procured from Nomura Siam International Co., Ltd. (Bangkok, Thailand). The mice were housed in polycarbonate cages with sawdust bedding under standardized laboratory conditions, maintaining a 12 h light/dark cycle and a controlled temperature range of 22°C–24°C. They had *ad libitum* access to standard rodent chow and clean water. Prior to experimentation, the mice underwent a 7 day acclimatization period to adjust to the laboratory environment [7, 8]. For euthanasia, CO_2_ inhalation was used as the primary method. The CO_2_ flow rate was regulated to gradually displace 30%–70% of the chamber volume/minute, minimizing animal distress by ensuring a smooth transition to unconsciousness. After CO_2_ exposure, cervical dislocation was performed as a secondary measure to confirm death. All experimental procedures involving animals were ethically reviewed and approved by the Animal Ethics Committee and were conducted in strict accordance with the Guide for the Care and Use of Laboratory Animals.

### 2.3. Rodent Malaria Parasite

The rodent malaria parasite *Plasmodium berghei* ANKA (PbANKA) strain was obtained from the Malaria Research and Reference Reagent Resource Center (MR4, Manassas, VA, USA). This parasite strain is commonly used in experimental malaria research due to its well-characterized virulence and pathogenesis. To ensure long-term viability and availability, the parasite was cryopreserved in liquid nitrogen using a glycerol-based cryoprotective solution. Periodically, the cryopreserved stock was thawed and passaged through mice via intraperitoneal (IP) injection of infected erythrocytes to maintain virulence and genetic stability. Parasitemia levels were monitored daily by Giemsa-stained blood smears, and parasitemia (%) was calculated using the formula:

(1) $$\begin{array}{c} \text{Parasitemia}\left(\%\right)=\frac{\text{Number of infected erythrocytes}\times 100}{\text{Total number of erythrocytes}}. \end{array}$$

An inoculum was prepared using 1 mL of infected blood obtained from a donor mouse with 10%–20% parasitemia via cardiac puncture. The blood was diluted to contain 1 × 10^7^ PbANKA-infected erythrocytes in 0.2 mL of 0.9% sodium chloride solution and inoculated by IP injection into naïve mice [7, 8].

### 2.4. Acute Toxicity Test

The acute toxicity of GGE was evaluated following OECD guidelines for acute oral toxicity testing by administering a single dose of 3000 mg/kg of GGE to a group of mice (*n* = 5) via oral gavage [9]. Control groups received an equivalent volume of DW under the same conditions. The mice were then observed for 14 days for signs of toxicity, including changes in behavior, physical appearance, and mortality. At the end of the observation period, surviving mice were sacrificed and blood samples were collected via cardiac puncture under anesthesia for hematological and biochemical analyses.

### 2.5. Determination of Hematological and Biochemical Parameters

Blood samples were collected from surviving mice via cardiac puncture under anesthesia. The blood was divided into two portions: one for hematological analysis and the other for biochemical analysis. For hematological analysis, blood samples were collected into tubes containing ethylenediaminetetraacetic acid (EDTA) as an anticoagulant. Hematological parameters were determined using an automated hematology analyzer (Sysmex XE-2100). The complete blood count (CBC) included measurements of red blood cell (RBC) count, white blood cell (WBC) count, hemoglobin (Hb) concentration, hematocrit (Hct), and platelet count. For biochemical analysis, blood samples were collected into tubes containing heparin as an anticoagulant. The plasma was separated by centrifugation and analyzed using an automated biochemical analyzer (Roche Cobas c311). The biochemical parameters measured included aspartate aminotransferase (AST), alanine aminotransferase (ALT), and alkaline phosphatase (ALP) to evaluate liver function, and blood urea nitrogen (BUN) and creatinine to assess kidney function.

### 2.6. Suppressive Antimalarial Test

The suppressive test, also known as the 4-day suppressive test, was conducted to evaluate the antimalarial activity of GGE [10]. Male ICR mice, aged 4–6 weeks and weighing 20–25 g, were infected with 1 × 10^7^ parasitized erythrocytes of PbANKA by IP injection and randomly divided into groups (*n* = 5 per group). After 2 h postinfection, the treatment groups received the GGE orally at doses of 100 mg/kg, 200 mg/kg, and 400 mg/kg once daily for 4 consecutive days. A negative control group received an equivalent volume of the DW, while a positive control group received chloroquine (CQ) at a dose of 10 mg/kg orally. On the fifth day, thin blood smears were prepared from tail blood, stained with Giemsa, and examined under a microscope to determine parasitemia. The percent inhibition of parasitemia was calculated to assess the efficacy of the extract in suppressing the parasite. The percent inhibition was determined using the following formula:

(2) $$\begin{array}{c} \text{inhibition}\left(\%\right)=\frac{\left(\text{parasitemia in negative control}–\text{parasitemia in treated group}\right)\times 100}{\text{parasitemia in negative control}}. \end{array}$$

Additionally, the mean survival time (MST) of the mice was recorded over an observation period of 30 days.

### 2.7. Curative Antimalarial Test

The curative antimalarial activity of the GGE was evaluated following established protocols [11]. Male ICR mice, aged 4–6 weeks and weighing 20–25 g, were infected with a 1 × 10^7^ PbANKA-infected erythrocytes via IP injection to induce malaria infection. After confirmation of parasitemia (typically around 5%–7% on Day 3 postinfection), the infected mice were randomly divided into treatment groups and administered the GGE at doses of 100 mg/kg, 200 mg/kg, and 400 mg/kg via oral gavage. Treatment was initiated on Day 3 postinfection and continued once daily for 4 days. Standard antimalarial drug (CQ) at 10 mg/kg and DW-treated groups served as positive and negative controls, respectively. Parasitemia levels were assessed daily using Giemsa-stained blood smears obtained from tail blood, followed by calculation of percent inhibition. Furthermore, MST was evaluated over a 30-day observation period.

### 2.8. Prophylactic Antimalarial Test

The prophylactic antimalarial activity of the GGE was evaluated using previously described protocol [12]. Male ICR mice, aged 4–6 weeks and weighing 20–25 g, were randomly divided into treatment groups and administered the extract at doses of 100 mg/kg, 200 mg/kg, and 400 mg/kg via oral gavage once daily for 4 days prior to infection. Following treatment, mice were infected with 1 × 10^7^ PbANKA-infected erythrocytes via IP injection. Parasitemia levels were monitored daily by Giemsa-stained blood smears from tail blood for 4 days, and percent inhibition was then calculated. Additionally, MST was recorded for a 30-day observation period to evaluate the protective efficacy of the GGE against malaria-induced mortality. CQ at 10 mg/kg and DW-treated groups served as positive and negative controls, respectively, to compare the prophylactic activity of the plant extract.

### 2.9. Determination of Mean Survival Time

The MST of the treated and control mice was determined over an observation period of 30 days [13]. The number of surviving mice in each group was recorded daily. The MST was calculated using the following formula:

(3) $$\begin{array}{c} \text{MST}\left(\text{day}\right)=\frac{\sum \left(\text{day of death}\times \text{number of mice that die on that day}\right)}{\text{total number of mice}}. \end{array}$$

### 2.10. Statistical Analysis

Data were expressed as mean ± standard error of the mean (SEM). Statistical analysis was performed using GraphPad Prism software (GraphPad Software, San Diego, CA, USA). Comparisons between groups were made using one-way analysis of variance (ANOVA) followed by post-hoc tests (Tukey's multiple comparisons test) to determine significant differences. A *p* value of less than 0.05 was considered statistically significant.

## 3. Results

### 3.1. Acute Toxicity of GGE in Mice

The acute toxicity of GGE was assessed in ICR mice following a single oral dose of 3000 mg/kg, with a 14-day observation period. Hematological and biochemical parameters were measured to evaluate potential toxic effects. Throughout the 14-day observation period, no significant behavioral changes were observed in the treated mice compared to the control group. The mice displayed normal activity levels, grooming behavior, and no signs of distress or discomfort. Additionally, there were no signs of toxicity such as tremors, convulsions, or abnormal posture. Importantly, no mortality was recorded in either the control or treated groups during the study period.

**Figure 2:**
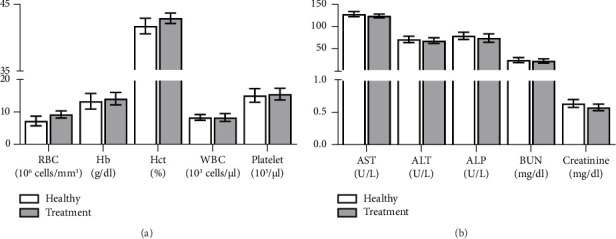
Acute toxicity test of GGE in ICR mice. Mice were administered a single dose of 3000 mg/kg of the extract and observed over a 14-day period. At the end of the observation period, mice were sacrificed, and blood samples were collected via cardiac puncture. (a) Hematological and (b) biochemical parameters were measured. Data are presented as mean ± SEM (*n* = 5). ALP, alkaline phosphatase; ALT, alanine aminotransferase; AST, aspartate aminotransferase; BUN, blood urea nitrogen.

The hematological analysis showed no significant differences in RBC count, Hb levels, and Hct values between the control and treated groups. WBC and platelet count also remained within normal ranges, indicating no adverse effects on these hematological indices (Figure 2(a)). Biochemical analysis revealed that the levels of AST, ALT, and ALP in the treated group were comparable to those of the control group, suggesting no hepatotoxicity induced by the extract. Similarly, BUN and creatinine levels, which are indicators of renal function, showed no significant differences between the control and treated groups (Figure 2(b)). Overall, the single dose of 3000 mg/kg of GGE did not result in any significant alterations in hematological or biochemical parameters, nor did it induce any behavioral changes, signs of toxicity, or mortality. These findings indicate that GGE is safe at the tested dose in this model.

### 3.2. Suppressive Antimalarial Efficacy of GGE Against PbANKA-Infected Mice

**Figure 3:**
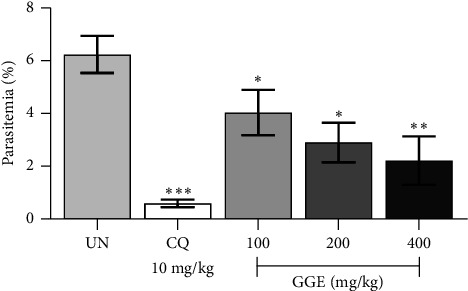
Suppressive antimalarial efficacy of GGE against PbANKA-infected mice. ICR mice were infected with PbANKA via IP injection and subsequently treated with GGE at doses of 100, 200, and 400 mg/kg or with CQ at 10 mg/kg, administered orally by gavage for 4 days. Parasitemia levels were measured. Data are presented as mean ± SEM. ⁣^∗^*p* < 0.05, ⁣^∗∗^*p* < 0.01, and ⁣^∗∗∗^*p* < 0.001 compared to the UN. CQ, chloroquine; GGE, *Gnetum gnemon* extract; UN, untreated group.

The suppressive antimalarial activity of GGE was evaluated in ICR mice infected with 1 × 10^7^ PbANKA-infected erythrocytes and treated with GGE at doses of 100, 200, and 400 mg/kg, as well as with CQ at 10 mg/kg, for 4 consecutive days.  Figure 3 showed that the parasitemia levels were significantly (*p* < 0.05) reduced in all GGE-treated groups compared to the untreated control group. Specifically, GGE at 100, 200, and 400 mg/kg showed a dose-dependent suppression of parasitemia with the inhibition of 34.92%, 53.02%, and 63.97%, respectively. The highest dose of GGE (400 mg/kg) exhibited the most pronounced antimalarial effect, with parasitemia levels significantly (*p* < 0.01) lower than those observed in the untreated group, indicating a strong suppressive effect of GGE on PbANKA infection. The standard antimalarial drug, CQ at 10 mg/kg, served as a positive control and demonstrated highly significant (*p* < 0.001) suppression with 89.68% inhibition compared to the untreated group. Moreover, changes in weight, body temperature, and packed cell volume were indeed monitored as part of our investigation. Our findings showed that GGE helped maintain these indicators within normal ranges, suggesting its potential to protect against malaria-induced weight loss, hypothermia, and anemia (data not shown). Overall, the results indicate that GGE possesses significant suppressive antimalarial activity in a dose-dependent manner, demonstrating its potential as a therapeutic agent against malaria.

Additionally, GGE showed a dose-dependent increase in MST, indicating improved survival with higher doses. The highest dose at 400 mg/kg significantly (*p* < 0.001) increased MST to 25.4 days, although not as effectively as the positive control, CQ, which resulted in an MST of 29.2 days (Table 1).

### 3.3. Curative Antimalarial Efficacy of GGE Against PbANKA-Infected Mice

**Figure 4:**
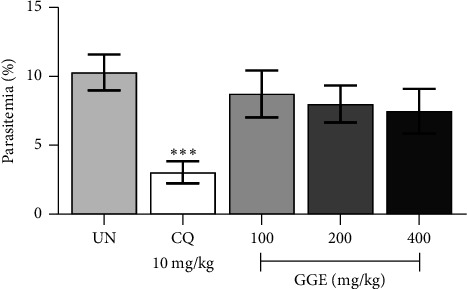
Curative antimalarial efficacy of GGE against PbANKA-infected mice. ICR mice were infected with PbANKA via IP injection for 4 days and treated with GGE at doses of 100, 200, and 400 mg/kg or with CQ at 10 mg/kg, administered orally by gavage for 4 days. Parasitemia levels were measured. Data are presented as mean ± SEM. ⁣^∗∗∗^*p* < 0.001 compared to the UN. CQ, chloroquine; GGE, *Gnetum gnemon* extract; UN, untreated group.

The curative antimalarial activity of GGE was evaluated in ICR mice infected with 1 × 10^7^ PbANKA-infected erythrocytes. Mice were treated with GGE at doses of 100, 200, and 400 mg/kg, administered orally by gavage for 4 consecutive days, starting from the day of infection. A control group was treated with CQ at a dose of 10 mg/kg, serving as a positive control. As indicated in Figure 4, treatment with CQ resulted in a significant reduction in parasitemia levels, with a 69.62% inhibition of parasitemia. This significant reduction (*p* < 0.001) underscores the strong curative antimalarial activity of CQ, a well-established antimalarial drug. The substantial inhibition of parasitemia by CQ confirms its effectiveness in treating established malaria infection and serves as a benchmark for evaluating the efficacy of other potential antimalarial agents. In contrast, none of the GGE-treated groups (100, 200, and 400 mg/kg) showed significant differences in parasitemia levels compared to the untreated control group. The lack of significant parasitemia reduction in the GGE-treated groups suggests that GGE, at the doses tested, does not exhibit strong curative antimalarial effects. Moreover, there were no significant differences in MST between the GGE-treated groups and the untreated control, with MSTs ranging from 10.4 to 11.0 days. The positive control (CQ) significantly (*p* < 0.001) extended MST to 27.0 days, demonstrating its efficacy in treating established infection (Table 1).

### 3.4. Prophylactic Antimalarial Efficacy of GGE Against PbANKA-Infected Mice

**Figure 5:**
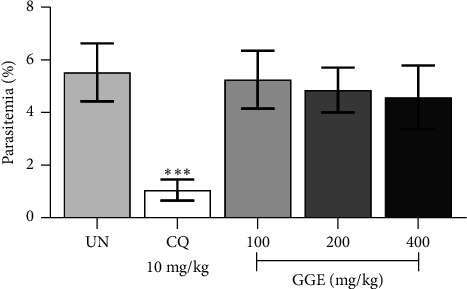
Prophylactic antimalarial efficacy of GGE against PbANKA-infected mice. ICR mice were treated with GGE at doses of 100, 200, and 400 mg/kg or with CQ at 10 mg/kg, administered orally by gavage for 4 days, and subsequently infected with PbANKA via IP injection. Parasitemia levels were measured. Data are presented as mean ± SEM. ⁣^∗∗∗^*p* < 0.001 compared to the UN. CQ, chloroquine; GGE, *Gnetum gnemon* extract; UN, untreated group.

The prophylactic antimalarial activity of GGE was evaluated in ICR mice infected with 1 × 10^7^ PbANKA-infected erythrocytes. Mice were pretreated with GGE at doses of 100, 200, and 400 mg/kg, administered orally by gavage for 4 consecutive days prior to infection. A control group was treated with CQ at a dose of 10 mg/kg, serving as a positive control. The results from Figure 5 indicated that CQ pretreatment significantly reduced parasitemia levels in mice infected with PbANKA. Specifically, CQ treatment achieved a 79.96% inhibition of parasitemia, demonstrating its potent prophylactic antimalarial activity. This significant reduction (*p* < 0.001) highlights the effectiveness of CQ as a standard antimalarial agent in preventing the establishment of infection. In contrast, pretreatment with GGE at doses of 100, 200, and 400 mg/kg did not result in significant reductions in parasitemia levels compared to the untreated control group. The lack of significant prophylactic effect at all tested doses of GGE suggests that, unlike CQ, the extract does not possess strong preventive properties against malaria infection in this model. Moreover, pretreatment with GGE, at the doses tested, did not provide significant increase in MST compared to the untreated control group. However, CQ pretreatment resulted in a much higher MST of 25.8 days, showcasing its strong prophylactic efficacy (Table 1).

## 4. Discussion

The study focused on evaluating the antimalarial properties of GGE against PbANKA infection in mice, exploring its suppressive, curative, and prophylactic potentials. The decision to use an in vivo model is supported by several scientific considerations. In vitro studies, though useful, cannot fully capture the pharmacological behavior of bioactive compounds, including their absorption, metabolism, bioavailability, and interactions within a living organism. An in vivo model provides a more comprehensive evaluation of efficacy, toxicity, and therapeutic potential, allowing for the study of effects on parasitemia, immune response, and survival outcomes [14]. Furthermore, plant extracts often exhibit synergistic interactions between multiple compounds that may not be accurately replicated in vitro. In vivo studies allow for the assessment of such interactions within the complex biological environment where they naturally occur. This is particularly important for antimalarial therapies, which may involve multiple biochemical pathways and immune mechanisms [15]. The use of *P. berghei* in our study is justified by its widespread acceptance as a reliable rodent malaria model for preclinical research. This model provides meaningful insights into the progression of malaria and treatment outcomes, helping us evaluate the potential of *G. gnemon* as a candidate for further pharmacological development [16, 17].

GGE was prepared by the hot water extraction combined with microwave-assisted heating for several reasons. First, hot water extraction aligns with the traditional preparation methods used for medicinal plants, including *G. gnemon*, where water-based decoctions are often used to extract bioactive compounds. This method ensures that the results are not only scientifically valid but also relevant to ethnopharmacological practices [18, 19]. Additionally, the choice of microwave-assisted extraction offers several advantages, such as enhanced efficiency, reduced extraction time, and minimal solvent use, aligning with green chemistry principles [20]. This technique also helps preserve heat-sensitive secondary metabolites that contribute to the antimalarial activity of the plant, such as flavonoids, tannins, and polyphenols [21]. Water-based extraction ensures that the final extract is nontoxic and suitable for biological testing, further supporting its use in pharmacological research.

The results reveal significant findings regarding the antimalarial efficacy and safety profile of GGE. The results of the acute toxicity test indicate that GGE is safe at a high dose of 3000 mg/kg. The absence of behavioral changes, signs of toxicity, and mortality, along with normal hematological and biochemical parameters, support the nontoxic nature of GGE in the acute setting.

The suppressive antimalarial activity of GGE was notable, with doses of 100, 200, and 400 mg/kg leading to significant reductions in parasitemia levels, achieving inhibition rates of 34.92%, 53.02%, and 63.97%, respectively. The highest dose tested (400 mg/kg) demonstrated substantial suppressive effects, although not as potent as the standard antimalarial drug, CQ, which showed an 89.68% inhibition. According to standard criteria, an extract is considered to exhibit significant antimalarial activity if it achieves a parasitemia inhibition of 30% or more [22]. In this study, the inhibition observed with the doses of GGE far exceeds this threshold, highlighting its potential as an effective antimalarial agent. The antimalarial activity of GGE can be attributed to its rich phytochemical profile, which includes compounds such as flavonoids, tannins, and phenolic acids [4]. Kaempferol, a flavonoid identified in *G. gnemon*, has been reported to exhibit significant antimalarial activity [23, 24]. Research indicates that kaempferol can inhibit the growth of *Plasmodium* parasites by disrupting the parasite's mitochondrial function and inhibiting protein synthesis [25, 26]. Similarly, gallic acid, another compound found in GGE, has demonstrated antimalarial properties. Gallic acid is known to induce oxidative stress in *Plasmodium* parasites, leading to cell death [27]. It has also been observed to interfere detoxification process of parasite by inhibiting hemozoin formation, resulting in the accumulation of toxic free heme within the parasite and ultimately leading to its death [28]. Additionally, in vitro studies have shown that the ethanol extract of *G. gnemon* leaves has an IC_50_ value of 29.4 *μ*g/mL against the CQ-sensitive strain of *P. falciparum* (3D7). Further investigations isolated several compounds from the leaves, with ursolic acid demonstrating the highest potency, showing IC_50_ values of 4.0 *μ*g/mL against the CQ-sensitive strain and 6.0 *μ*g/mL against the resistant strain (Dd2). Other compounds, such as 2,3-dihydroxypropyl icosanoate and oleic acid, also exhibited moderate antimalarial activity, with IC_50_ values ranging from 9.5 to 21.1 *μ*g/mL [5]. Hence, the suppressive antimalarial activity of GGE might be attributed to the presence of these active compounds.

Despite its encouraging suppressive effects, GGE did not show notable curative benefits. The parasitemia levels in the groups treated with GGE were similar to those in the untreated control group, suggesting that GGE might not be effective in treating fully established malaria infections. This could be because the bioactive compounds in GGE, although effective in inhibiting the early stages of parasite development as seen in the suppressive test, might not be strong enough to clear existing infections or could be limited by the specific stage of parasite development [29]. The prophylactic efficacy of GGE was also limited, as pretreatment with GGE at the tested doses did not result in significant reductions in parasitemia levels compared to controls. The lack of significant prophylactic effects of GGE suggests that its bioactive compounds may not provide sufficient preemptive protection against malaria infection. The limited efficacy may also be attributed to factors such as the pharmacokinetics and bioavailability of the active compounds in GGE. If the bioavailability is low, even potent compounds may not reach therapeutic levels [30, 31].

A recent study has evaluated the antimalarial effects of *Coriandrum sativum* using similar in vivo models with *P. berghei* infection, providing relevant insights for comparison [32]. The coriander extract showed significant antimalarial efficacy, achieving 82.74% parasitemia inhibition in the suppressive model at the highest dose (400 mg/kg) and 78.49% in the curative model, with improved survival outcomes. In contrast, our study on GGE achieved a maximum parasitemia reduction of 63.97% in the suppressive test but did not exhibit significant curative or prophylactic effects. Both extracts were found to be nontoxic, demonstrating their potential as safe treatment options. These findings suggest that GGE shows promise in suppressing malaria, highlighting the need for further exploration of plant-based therapies and potential combination treatments.

MST analysis further supports the findings, with GGE treatment resulting in improved survival rates during suppressive tests but not significantly affecting survival in curative and prophylactic tests. The highest dose of GGE (400 mg/kg) extended MST to 25.4 days, although this was still lower than the MST observed with CQ treatment, which reached 29.2 days. The improved survival rates observed during suppressive tests indicate that GGE has the potential to suppress parasitemia and prolong survival when administered concurrently with the malaria parasite inoculation. In addition, the lack of significant improvement in survival rates during curative and prophylactic tests suggests that GGE may have limited efficacy in treating established infections or preventing malaria infection altogether. Plant extract was considered active if it increased the survival days of treated infected mice compared to those of untreated infected mice [33]. Hence, the MST supports the potential of GGE in suppressing parasitemia and improving survival rates in malaria-infected individuals.

The strength of this study lies in its comprehensive evaluation of the antimalarial potential of GGE, using multiple models (suppressive, curative, and prophylactic) to assess efficacy against *P. berghei*. The dose-dependent analysis demonstrated a clear relationship between dose and parasitemia suppression, with the highest dose achieving 63.97% inhibition. Additionally, the extract showed no toxicity at high doses, supporting its potential for further investigation. However, the study has some limitations. The extract exhibited limited curative and prophylactic effects, restricting its use as a standalone treatment.

## 5. Conclusion

This study investigated the antimalarial properties of GGE in PbANKA-infected ICR mice, focusing on its suppressive, curative, and prophylactic activities. The findings reveal that GGE possesses significant suppressive antimalarial activity, as evidenced by the reduction in parasitemia levels and increased MST of treated mice. However, GGE did not demonstrate significant curative or prophylactic effects at the tested doses, unlike the positive control CQ, which showed robust efficacy in both curative and prophylactic tests. The absence of acute toxicity at a high dose of 3000 mg/kg indicates the safety of GGE for further investigations. Despite its promising suppressive activity, the lack of significant curative and prophylactic effects suggests that GGE alone may not be sufficient as a comprehensive antimalarial treatment. Future studies should explore the potential of GGE in combination therapies, evaluate different dosages, and identify specific bioactive compounds responsible for its antimalarial activity. Overall, this research highlights the potential of *Gnetum gnemon* as a source of antimalarial agents and underscores the importance of continued exploration of natural products for the development of new antimalarial therapies. Further in vivo and clinical studies are warranted to fully understand the therapeutic potential and mechanisms of action of GGE in malaria treatment.

## Figures and Tables

**Table 1:** MST of GGE in suppressive, curative, and prophylactic antimalarial tests.

Antimalarial test	Treatment	MST (day)
Suppressive	UN	10.0 ± 1.58
	100 mg/kg of GGE	16.6 ± 1.52⁣^∗^
	200 mg/kg of GGE	22.0 ± 1.58⁣^∗∗^
	400 mg/kg of GGE	25.4 ± 1.14⁣^∗∗∗^
	10 mg/kg of CQ	29.2 ± 0.84⁣^∗∗∗^

Curative	UN	10.2 ± 1.92
	100 mg/kg of GGE	10.4 ± 1.14
	200 mg/kg of GGE	11.0 ± 1.58
	400 mg/kg of GGE	10.6 ± 1.52
	10 mg/kg of CQ	27.0 ± 1.58⁣^∗∗∗^

Prophylactic	UN	10.4 ± 1.14
	100 mg/kg of GGE	11.2 ± 1.30
	200 mg/kg of GGE	11.6 ± 1.52
	400 mg/kg of GGE	10.6 ± 2.19
	10 mg/kg of CQ	25.8 ± 2.59⁣^∗∗∗^

Abbreviations: CQ, chloroquine; GGE, *Gnetum gnemon* extract; UN, untreated group.

⁣^∗^*p* < 0.05.

⁣^∗∗^*p* < 0.01.

⁣^∗∗∗^*p* < 0.001 compared to UN.

## Data Availability

The data that support the findings of this study are openly available in Figshare at https://figshare.com/s/69cceec8f6104b85596c, reference number 10.6084/m9.figshare.26197844.
